# A survey of prevalence and phenotypic and genotypic assessment of antibiotic resistance in *Staphylococcus aureus* bacteria isolated from ready-to-eat food samples collected from Tehran Province, Iran

**DOI:** 10.1186/s41182-021-00366-4

**Published:** 2021-10-11

**Authors:** Arash Mesbah, Zohreh Mashak, Zohreh Abdolmaleki

**Affiliations:** 1 Faculty of Veterinary Medicine, Karaj Branch, Islamic Azad University, Karaj, Iran; 2 Department of Food Hygiene, Karaj Branch, Islamic Azad University, Karaj, Iran; 3 Department of Pharmacology, Karaj Branch, Islamic Azad University, Karaj, Iran

**Keywords:** *Staphylococcus aureus*, Prevalence, Phenotype of antibiotic resistance, Genotype of antibiotic resistance, Ready-to-eat food

## Abstract

**Background:**

Resistant *Staphylococcus aureus* (*S. aureus*) bacteria are considered among the major causes of foodborne diseases. This survey aims to assess genotypic and phenotypic profiles of antibiotic resistance in *S. aureus* bacteria isolated from ready-to-eat food samples.

**Methods:**

According to the previously reported prevalence of *S. aureus* in ready-to-eat food samples, a total of 415 ready-to-eat food samples were collected from Tehran province, Iran. *S. aureus* bacteria were identified using culture and biochemical tests. Besides, the phenotypic antibiotic resistance profile was determined by disk diffusion. In addition, the genotypic pattern of antibiotic resistance was determined using the PCR.

**Results:**

A total of 64 out of 415 (15.42%) ready-to-eat food samples were contaminated with *S. aureus*. Grilled mushrooms and salad olivieh harbored the highest contamination rate of (30%), while salami samples harbored the lowest contamination rate of 3.33%. In addition, *S. aureus* bacteria harbored the highest prevalence of resistance to penicillin (85.93%), tetracycline (85.93%), gentamicin (73.43%), erythromycin (53.12%), trimethoprim-sulfamethoxazole (51.56%), and ciprofloxacin (50%). However, all isolates were resistant to at least four antibiotic agents. Accordingly, the prevalence of *tetK* (70.31%), *blaZ* (64.06%), *aacA-D* (57.81%), *gyrA* (50%), and *ermA* (39.06%) was higher than that of other detected antibiotic resistance genes. Besides, *AacA-D* + *blaZ* (48.43%), *tetK* + *blaZ* (46.87%), *aacA-D* + *tetK* (39.06%), *aacA-D* + *gyrA* (20.31%), and *ermA* + *blaZ* (20.31%) were the most frequently identified combined genotypic patterns of antibiotic resistance.

**Conclusion:**

Ready-to-eat food samples may be sources of resistant *S. aureus,* which pose a hygienic threat in case of their consumption. However, further investigations are required to identify additional epidemiological features of *S. aureus* in ready-to-eat foods.

## Background

Ready-to-eat food samples are among the most popular foodstuffs among Iranian people. Diverse kinds of ready-to-eat food samples, particularly hamburgers, chicken nuggets, salad olivieh (chicken meat-based salad with eggs and potatoes), salami, falafel (pea-based food with high amounts of different spices), grilled mushrooms, and Mexican corn are presented as street foods in Iran. Use of low-quality raw materials and poor hygienic conditions in preparation of these foodstuffs cause microbial contamination [1, 2].

*Staphylococcus aureus* (*S. aureus*), i.e. a Gram-positive and catalase-positive bacterium, is a major cause of food-borne diseases with a short incubation period as well as symptoms, such as weakness, vomiting, nausea, and abdominal cramps in people [3, 4]. Contaminated foodstuffs, particularly ready-to-eat food samples, are considered reservoirs of *S. aureus* [5, 6].

This bacterium develops resistance to diverse kinds of antibiotic agents [7]. Resistant *S. aureus* bacteria are responsible for about 100,000 infectious disease cases, with about an annual mortality rate of 20–30% in the United States [8]. Resistant *S. aureus* bacteria cause complicated diseases for a long period [9]. Research reports that *S. aureus* bacteria harbor high resistance to diverse kinds of antibiotic drugs, particularly penicillins, cephalosporins, tetracyclines, aminoglycosides, macrolides, and fluoroquinolones [7, 10].

Some antibiotic resistance genes are responsible for development of antibiotic resistance in *S. aureus* strains [11]. *TetK* and *tetM* (tetracycline resistance genes), *ermA* and *msrA* (macrolide resistance genes), *gyrA* and *grlA* (fluoroquinolone resistance genes), *blaZ* (penicillin resistance gene), *dfrA* (folate inhibitor resistance gene), *rpoB* (ansamycin resistance gene), *aacA-D* (aminoglycoside resistance gene), *linA* (lincosamide resistance gene), and *cat1* (phenicol resistance gene) are the major resistance genes among *S. aureus* bacteria [11].

Given the high consumption rate of ready-to-eat foodstuffs in Iran and the high importance of *S. aureus* as a food-borne pathogen, the present survey was performed to assess the prevalence as well as phenotypic and genotypic patterns of antibiotic resistance in *S. aureus* bacteria isolated from diverse kinds of ready-to-eat food samples.

## Results

Table 1 shows the prevalence of *S. aureus* in diverse kinds of ready-to-eat food samples. A total of 64 out of 415 (15.42%) ready-to-eat food samples were contaminated with *S. aureus*. Accordingly, grilled mushrooms (30%) and salad olivieh (30%) were the most commonly contaminated samples. In contrast, the lowest prevalence of *S. aureus* was found in salami samples (3.33%). A statistically significant difference was observed between different types of ready-to-eat food samples and *S. aureus* prevalence (*P* < 0.05).

**Table 1:** Prevalence of *S. aureus* in diverse kinds of ready-to-eat food samples

Types of samples	No. of samples collected	No. of samples positive for *S. aureus* (%)
Hamburgers	75	7 (9.33)
Chicken nuggets	70	5 (7.14)
Salad olivieh	60	18 (30)
Salami	60	2 (3.33)
Felafel	50	10 (20)
Grilled mushrooms	50	15 (30)
Mexican corn	50	7 (14)
Total	415	64 (15.42)

Table 2 shows the phenotypic profile of antibiotic resistance in *S. aureus* strains isolated from diverse kinds of ready-to-eat food samples. Accordingly, *S. aureus* isolates harbored the highest prevalence of resistance to the antibiotics of penicillin (85.93%), tetracycline (85.93%), gentamicin (73.43%), erythromycin (53.12%), trimethoprim-sulfamethoxazole (51.56%), and ciprofloxacin (50%). However, the lowest prevalence of resistance was found to the antibiotic agents of rifampin (26.56%), doxycycline (26.56%), and chloramphenicol (28.12%). The prevalence of resistance to the antibiotic agents of amikacin, azithromycin, levofloxacin, and clindamycin was 35.93%, 42.18%, 37.50%, and 37.50%, respectively. Accordingly, a statistically significant difference was observed between various types of ready-to-eat food samples and prevalence of antibiotic resistance (*P* < 0.05). Moreover, significant differences were observed in the prevalence of resistance between antibiotic agents of gentamicin and amikacin (*P* < 0.05), azithromycin and erythromycin (*P* < 0.05), tetracycline and doxycycline (*P* < 0.05), as well as ciprofloxacin and levofloxacin (*P* < 0.05).

**Table 2:** Phenotypic profile of antibiotic resistance in *S. aureus* isolates recovered from diverse kinds of ready-to-eat food samples

Origins (*N* *S. aureus*)	*N* (%) isolates resistant to each antibiotic												
	Penicillins	Aminoglycosides		Macrolides		Tetracyclines		Fluoroquinolones		Lincosamides	Folate inhibitors	Phenicols	Ansamycins
	P10^*^	Gen	Amk	Azi	Ert	Tet	Dox	Cip	Lev	Clin	Tr-Sul	C30	Rif
Hamburgers (7)	6 (85.71)	5 (71.42)	2 (28.57)	3 (42.85)	4 (57.14)	6 (85.71)	2 (28.57)	4 (57.14)	3 (42.85)	3 (42.85)	4 (57.14)	1 (14.28)	2 (28.57)
Chicken nuggets (5)	5 (100)	4 (80)	2 (40)	3 (60)	4 (80)	5 (100)	2 (40)	3 (60)	3 (60)	3 (60)	3 (60)	4 (80)	2 (40)
Salad olivieh (18)	15 (83.33)	14 (77.77)	8 (44.44)	6 (33.33)	9 (50)	16 (88.88)	5 (27.77)	9 (50)	5 (27.77)	5 (27.77)	9 (50)	3 (16.66)	3 (16.66)
Salami (2)	2 (100)	1 (50)	–	1 (50)	1 (50)	2 (100)	–	1 (50)	1 (50)	1 (50)	1 (50)	–	–
Felafel (10)	8 (80)	7 (70)	4 (40)	4 (40)	5 (50)	8 (80)	3 (30)	4 (40)	3 (30)	4 (40)	5 (50)	3 (30)	3 (30)
Grilled mushrooms (15)	13 (86.66)	11 (73.33)	5 (33.33)	6 (40)	7 (46.66)	13 (86.66)	3 (20)	7 (46.66)	5 (33.33)	5 (33.33)	7 (46.66)	5 (33.33)	5 (33.33)
Mexican corn (7)	6 (85.71)	5 (71.42)	2 (28.57)	4 (57.14)	4 (57.14)	5 (71.42)	2 (28.57)	4 (57.14)	4 (57.14)	3 (42.85)	4 (57.14)	2 (28.57)	2 (28.57)
Total (64)	55 (85.93)	47 (73.43)	23 (35.93)	27 (42.18)	34 (53.12)	55 (85.93)	17 (26.56)	32 (50)	24 (37.50)	24 (37.50)	33 (51.56)	18 (28.12)	17 (26.56)

*P10: penicillin (10 µg/disk), Gen: gentamicin (10 µg/disk), Amk: amikacin (30 µg/disk), Azi: azithromycin (15 µg/disk), Ert: erythromycin (15 µg/disk), Tet: tetracycline (30 µg/disk), Do: doxycycline (30 µg/disk), Cip: ciprofloxacin (5 µg/disk), Lev: levofloxacin (5 µg/disk), Clin: clindamycin (2 µg/disk), Tr-Sul: trimethoprim-sulfamethoxazole (25 µg/disk), C30: chloramphenicol (30 µg/disk), Rif: rifampin (5 µg/disk)

**Fig. 1:**
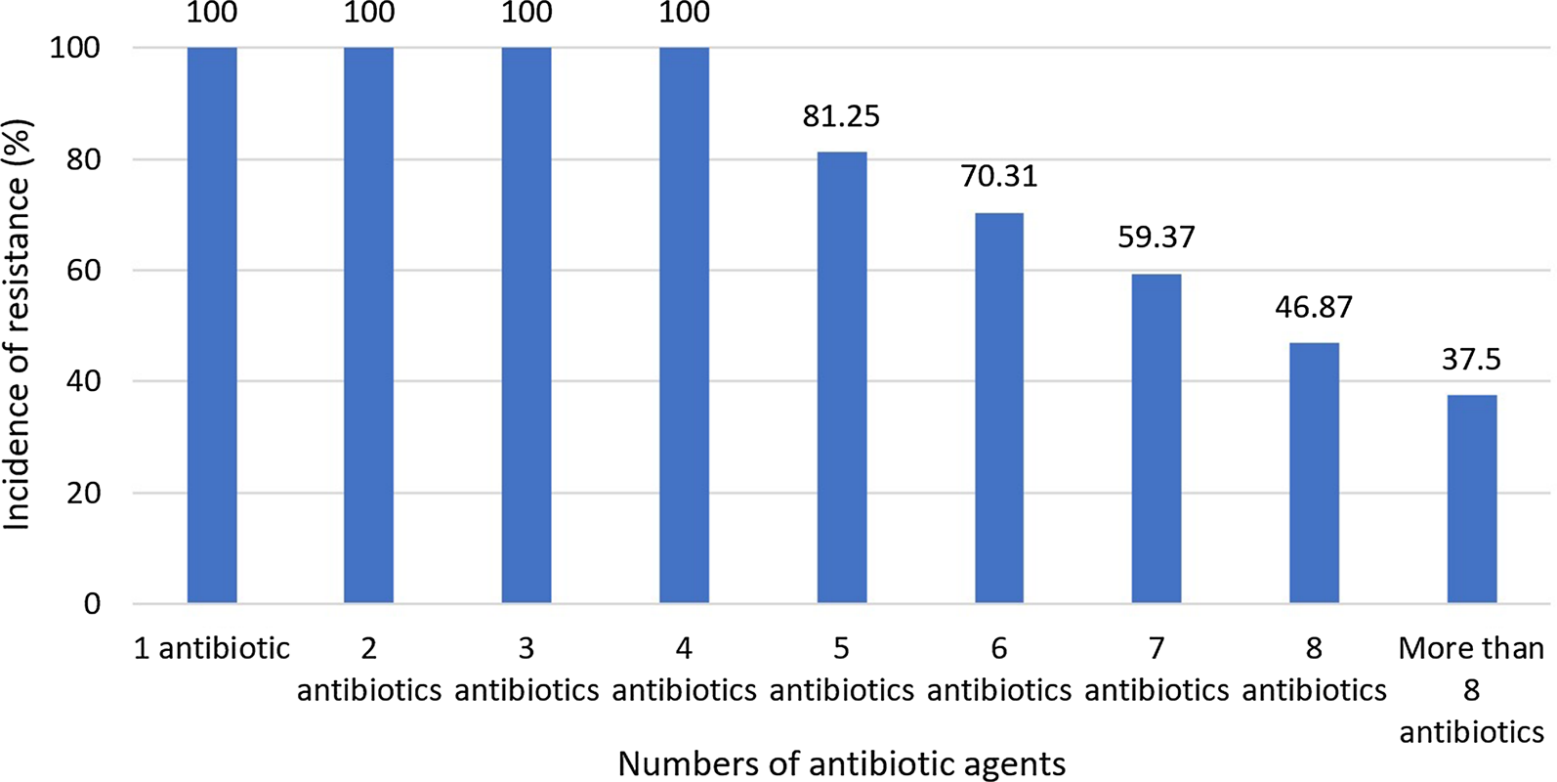
Prevalence of multi-drug resistant *S. aureus* bacteria isolated from ready-to-eat food samples; the results were interpreted from a total of 64 *S. aureus* isolates

Figure 1 shows the prevalence of multi-drug resistant *S. aureus* strains isolated from ready-to-eat food samples. Accordingly, the prevalence of resistance to at least four antibiotic agents was 100%, while it amounted to 37.50% in more than eight antibiotic agents.

Table 3 shows the genotypic profile of antibiotic resistance in *S. aureus* strains isolated from diverse kinds of ready-to-eat food samples. According to the table, *TetK* (70.31%), *blaZ* (64.06%), *aacA-D* (57.81%), *gyrA* (50%), and *ermA* (39.06%) were the most frequently identified antibiotic resistance genes. Prevalence of *tetM* (10.93%), *grlA* (10.93%), *linA* (18.75%), and *rpoB* (18.75%) was lower than that of other antibiotic resistance genes. Besides, there was a statistically significant difference between various types of samples and prevalence of antibiotic resistance genes (*P* < 0.05). Furthermore, statistically significant differences were observed in the distribution of the antibiotic resistance genes of *tetK* and *tetM* (*P* < 0.05), *msrA* and *ermA* (*P* < 0.05), as well as *gyrA* and *grlA* (*P* < 0.05).

**Table 3:** Genotypic profile of antibiotic resistance in *S. aureus* isolates recovered from diverse kinds of ready-to-eat food samples

Origins (*N* *S. aureus*)	*N* (%) isolates harbored each antibiotic resistance gene											
	Penicillins	Aminoglycosides	Macrolides		Tetracyclines		Fluoroquinolones		Lincosamides	Folate inhibitors	Phenicols	Ansamycins
	*blaZ*	*aacA-D*	*msrA*	*ermA*	*tetK*	*tetM*	*gyrA*	*grlA*	*linA*	*dfrA*	*cat1*	*rpoB*
Hamburgers (7)	4 (57.14)	4 (57.14)	2 (28.57)	3 (42.85)	5 (71.42)	1 (28.57)	3 (42.85)	1 (28.57)	2 (28.57)	2 (28.57)	2 (28.57)	2 (28.57)
Chicken nuggets (5)	3 (60)	2 (40)	2 (40)	3 (60)	4 (80)	1 (40)	2 (40)	1 (40)	2 (40)	2 (40)	2 (40)	2 (40)
Salad olvieh (18)	13 (81.25)	12 (66.66)	4 (22.22)	5 (27.77)	14 (77.77)	3 (27.77)	6 (33.33)	2 (11.11)	3 (27.77)	4 (22.22)	3 (27.77)	2 (11.11)
Salami (2)	1 (50)	1 (50)	–	1 (50)	1 (50)	–	1 (50)	–	–	–	–	–
Felafel (10)	6 (60)	5 (50)	3 (30)	4 (40)	6 (60)	1 (30)	2 (20)	1 (10)	2 (20)	3 (30)	3 (30)	2 (20)
Grilled mushrooms (15)	10 (66.66)	9 (60)	4 (26.66)	5 (33.33)	11 (73.33)	1 (20)	5 (33.33)	1 (20)	2 (13.33)	2 (13.33)	2 (13.33)	3 (20)
Mexican corn (7)	4 (57.14)	4 (57.14)	2 (28.57)	4 (57.14)	4 (57.14)	–	2 (28.57)	1 (14.28)	1 (14.28)	2 (28.57)	2 (28.57)	1 (14.28)
Total (64)	41 (64.06)	37 (57.81)	17 (26.56)	25 (39.06)	45 (70.31)	7 (10.93)	21 (50)	7 (10.93)	12 (18.75)	15 (23.43)	14 (21.87)	12 (18.75)

Table 4 shows the combined genotypic profile of antibiotic resistance in *S. aureus* strains isolated from diverse kinds of ready-to-eat food samples. Accordingly, a total of 19 diverse combined genotypic patterns of antibiotic resistance were identified in the *S. aureus* isolates. Besides, *AacA-D* + *blaZ* (48.43%), *tetK* + *blaZ* (46.87%), *aacA-D* + *tetK* (39.06%), *aacA-D* + *ermA* (20.31%), *aacA-D* + *gyrA* (20.31%), and *ermA* + *blaZ* (20.31%) were the most frequently identified combined genotypic patterns of antibiotic resistance. In addition, prevalence of *msrA* + *gyrA* (3.12%), *ermA* + *rpoB* (3.12%), *tetK* + *rpoB* (4.68%), *aacA-D* + *rpoB* (6.25%), *blaZ* + *rpoB* (7.81%), *tetK* + *msrA* (10.93%), and *ermA* + *gyrA* (10.93%) was lower than that of other identified combined antibiotic resistance profiles. However, none of the *S. aureus* isolates harbored the combined *msrA* + *rpoB* genotypic pattern.

**Table 4:** Combined genotypic profile of antibiotic resistance in *S. aureus* isolates recovered from diverse kinds of ready-to-eat food samples

Origins (*N* *S. aureus*)	*N* (%) isolates harbored combined antibiotic resistance genes																		
	*aacA-D* + *tetK*	*aacA-D* + *ermA*	*aacA-D* + *msrA*	*aacA-D* + *blaZ*	*aacA-D* + *gyrA*	*aacA-D* + *rpoB*	*tetK* + *ermA*	*tetK* + *msrA*	*tetK* + *blaZ*	*tetK* + *gyrA*	*tetK* + *rpoB*	*ermA* + *blaZ*	*ermA* + *gyrA*	*ermA* + *rpoB*	*msrA* + *blaZ*	*msrA* + *gyrA*	*msrA* + *rpoB*	*blaZ* + *gyrA*	*blaZ* + *rpoB*
Hamburgers (7)	2 (28.57)	1 (28.57)	1 (28.57)	3 (42.85)	2 (28.57)	1 (28.57)	1 (28.57)	1 (28.57)	3 ()	1 (28.57)	–	1 (28.57)	1 (28.57)	v	1 ()	–	–	1 (28.57)	1 (28.57)
Chicken nuggets (5)	2 (40)	1 (20)	1 (20)	3 (60)	2 (40)	–	1 (20)	1 (20)	2 (40)	1 (20)	–	1 (20)	–	–	1 (20)	–	–	1 (20)	1 (20)
Salad olvieh (18)	9 (50)	3 (27.77)	2 (11.11)	10 (55.55)	3 (27.77)	1 (5.55)	2 (11.11)	1 (5.55)	10 (55.55)	4 (22.22)	1 (5.55)	3 (27.77)	2 (11.11)	1 (5.55)	3 (27.77)	1 (5.55)	–	2 (11.11)	1 (5.55)
Salami (2)	1 (50)	1 (50)	–	1 (50)	1 (50)	–	1 (50)	–	1 (50)	–	–	1 (50)	–	–	–	–	–	–	–
Felafel (10)	3 (30)	2 (20)	1 (10)	4 (40)	1 (10)	–	2 (20)	1 (10)	4 (40)	1 (10)	1 (10)	2 (20)	1 (10)	–	2 (20)	–	–	1 (10)	–
Grilled mushroom (15)	6 (40)	3 (20)	2 (13.33)	8 (53.33)	3 (20)	2 (13.33)	3 (20)	2 (13.33)	8 (53.33)	2 (13.33)	1 (6.66)	3 (20)	2 (13.33)	1 (6.66)	2 (13.33)	1 (6.66)	–	3 (20)	2 (13.33)
Mexican corn (7)	2 (28.57)	2 (28.57)	1 (14.28)	2 (28.57)	1 (14.28)	–	2 (28.57)	1 (14.28)	2 (28.57)	1 (14.28)	–	2 (28.57)	1 (14.28)	–	1 (14.28)	–	–	1 (14.28)	–
Total (64)	25 (39.06)	13 (20.31)	8 (12.50)	31 (48.43)	13 (20.31)	4 (6.25)	12 (18.75)	7 (10.93)	30 (46.87)	10 (15.62)	3 (4.68)	13 (20.31)	7 (10.93)	2 (3.12)	10 (15.62)	2 (3.12)	–	9 (14.06)	5 (7.81)

## Discussion

Contaminated ready-to-eat food samples, especially those of an animal origin, are considered probable causes of *S. aureus* transmission to the human population [12].

The present survey was performed to evaluate the prevalence as well as phenotypic and genotypic profiles of antibiotic resistance in *S. aureus* bacteria isolated from the samples of hamburgers, salami, grilled mushrooms, falafel, salad olivieh, chicken nuggets, and Mexican corn. The prevalence of *S. aureus* was 15.42% in the examined samples. Besides, a higher prevalence was observed in grilled mushrooms (30%) and salad olivieh (30%), while a lower prevalence was found in salami samples (3.33%). This finding could have been due to the different levels of water activity (*a*_w_) and pH values in diverse food samples. Furthermore, the use of low-quality and contaminated raw ingredients might be the reason for the high prevalence of bacteria in ready-to-eat food samples. However, the transmission of *S. aureus* from infected staff to food samples should be recognized as well. Foodstuff contamination with *S. aureus* may be directly caused by infected food animals, or their products, such as meat, or by poor hygiene throughout their processing. The low prevalence of *S. aureus* in salami samples could have been due to the high temperature used in their processing. A similar survey was conducted by Safarpoor Dehkordi et al. [13], in which they showed the prevalence of *S. aureus* in raw meat, raw chicken, grilled meat, grilled chicken, soup, salad, and rice samples collected from hospital kitchens was 26.31%, 27.02%, 16.12%, 8.53%, 6.38%, 7.14%, and 4.20%, respectively. Besides, they showed that food sample manipulation by the infected staff of hospital kitchens was the main causative factor for development of *S. aureus*. Hasanpour Dehkordi et al. [7] reported that the prevalence of *S. aureus* bacteria in diverse kinds of food samples was within the range of 10.00 and 24.00%. A high prevalence of *S. aureus* bacteria was also reported in diverse kinds of foodstuffs from the continents of Asia [12, 14], Europe [15–20], Africa [21], America [22, 23], and Australia [24]. In the same vein, Wu et al. [25] reported that the prevalence of *S. aureus* in raw meat, pork, beef, poultry and mutton, sausages, frozen meat, pork, beef, poultry, mutton, and dumpling, and ready-to-eat meat, pork, beef, poultry, and mutton samples were 51.00%, 47.70%, 50.40%, 67.90%, 54.50%, 18.60%, 43.30%, 50.00%, 31.40%, 60.90%, 30.90%, 29.40%, 12.20%, 12.70%, 25.00%, 11.80% and 0%, respectively. In addition, they reported that the prevalence of *S. aureus* in ready-to-eat food samples was relatively lower than that in raw products, which could have been due to processing operations, such as heating as well as the adding of additives to ready-to-eat food samples. Achi and Madubuike (2007) reported that the prevalence of *S. aureus* in ready-to-eat fish sausage, meat sausage, fried fish, fried meat, suya, moin moin, wash water, and rinse water samples was 23.60%, 29.70%, 8.30%, 6.60%, 17.20%, 13.40%, 27.60%, and 18.10%, respectively [26]. Similarly, they introduced water and ready-to-eat food samples as the sources of *S. aureus*.

Findings of the present survey showed a high prevalence of resistance to diverse classes of antibiotic agents, particularly penicillins, tetracyclines, aminoglycosides, macrolides, fluoroquinolones, phenicols, and ansamycins. Additionally, some strains harbored antibiotic resistance genes. Chloramphenicol is a forbidden antibiotic agent, which is sometimes used to treat infections in poultry. Emergence of resistance to this antibiotic may imply its unauthorized prescription. Besides, development of antibiotic resistance to chloramphenicol could indirectly imply the poultry-based origin of the isolated *S. aureus* bacteria. Most of the examined samples, particularly salad olivieh, chicken nuggets, and hamburgers were made from poultry meat and its products. Additionally, *S. aureus* bacteria isolated from salad olivieh, chicken nugget, and hamburger samples had a moderate prevalence of chloramphenicol resistance (16.66%, 80%, and 14.28%, respectively). Moreover, the prevalence of the *cat1* chloramphenicol encoding gene among the *S. aureus* bacteria isolated from salad olivieh, chicken nugget, and hamburger samples was high, having been 27.77%, 40%, and 28.57%, respectively. Thus, the findings could indirectly verify the origin of the *S. aureus* isolates.

A high prevalence of multi-drug resistant *S. aureus* harboring resistance to tetracyclines [13, 27–29], phenicols [13, 27, 28], penicillins [13, 27–30] macrolides [13, 27–30], aminoglycosides [13, 27–31], folate inhibitors [12, 25–29], lincosamides [13, 27–30], fluoroquinolones [13, 27–31], ansamycins [13, 27–29], and cephems [13, 27–30] was also established by diverse studies. Besides, the high prevalence of *linA*, *aacA-D*, *ermA* and *msrA*, *gyrA* and *grlA*, *blaZ*, *cat1*, *tetK* and *tetM*, *rpoB,* and *dfrA1* antibiotic resistance genes was reported in the current survey. Safarpoor Dehkordi et al. [13] reported that the prevalence of resistance of *S. aureus* bacteria isolated from processed food samples to penicillin, ceftaroline, gentamicin, amikacin, kanamycin, azithromycin, erythromycin, tetracycline, doxycycline, ciprofloxacin, levofloxacin, clindamycin, trimethoprim-sulfamethoxazole, chloramphenicol, and rifampin antibiotic agents was 100%, 10%, 81.08%, 70.27%, 43.24%, 59.45%, 86.48%, 100%, 81.08%, 48.64%, 43.24%, 48.64%, 83.78%, 29.72%, and 35.13%, respectively. Besides, they reported that the prevalence of *aacA-D*, *tetK*, *tetM*, *msrA*, *ermA*, *ermC*, and *linA* antibiotic resistance genes was 62.16%, 72.97%, 27.02%, 64.86%, 72.97%, 27.02%, and 43.24%, respectively. In contrast to our findings, they found that *vatA* (45.94%), *vatB* (18.91%), and *vatC* (5.40%) antibiotic resistance genes were among methicillin-resistant *Staphylococcus aureus* (MRSA) isolates. Besides, a higher prevalence of resistance to antibiotic agents was reported in their study because they assessed MRSA isolates, which according to them, harbored a higher prevalence of resistance. Fowoyo and Ogunbanwo [32] reported that the *S. aureus* bacteria isolated from ready-to-eat foodstuffs harbored a high prevalence of resistance to trimethoprim-sulfamethoxazole (74.90%), ampicillin (86.70%), cefotaxime (3.50%), amoxicillin-clavulanic acid (52.50%), ciprofloxacin (23.90%), oxacillin (35.70%), gentamicin (11.40%), erythromycin (15.70%), and ofloxacin (7.10%). Compared to the present research, they reported a higher prevalence of resistance to trimethoprim-sulfamethoxazole (74.90%), while a lower prevalence of resistance to erythromycin (15.70%), gentamicin (11.40%), and ciprofloxacin (23.90%) was reported by them. This finding could be due to the fact that they only assessed the antibiotic resistance pattern of the coagulase-negative staphylococcal genus. The relatively low prevalence of resistance to chloramphenicol (8.33%) may be due to its illegal and unselective prescription, especially in veterinary medicine [33, 34]. A high prevalence of *tetK*, *blaZ*, *aacA-D*, *gyrA,* and *ermA* antibiotic resistance genes was reported in Algeria [35], South Africa [36], China [37], and Taiwan [38]. Akanbi et al. [39] reported that *blaZ*, *mecA*, *rpoB*, *ermB,* and *tetM* were the most commonly identified antibiotic resistance genes among the *S. aureus* bacteria isolated from food samples in South Africa. A high distribution of *mecA*, *gyrA*, *grlA,* and *cfr* was reported in the *S. aureus* bacteria recovered from chicken meat in Egypt [40]. Consistent with the present survey, an Iranian survey [41] showed that oxacillin, gentamicin, penicillin, tetracycline, and erythromycin-resistant *S. aureus* bacteria, isolated from milk and dairy products, carried a high prevalence of *blaZ*, *aacA-aphD*, *mecA*, *tetK* and *tetM*, *ermB*, *ermA*, *ermT*, *ermC*, *msrB,* and *msrA* antibiotic resistance genes. In the same vein, a similar phenotypic profile of antibiotic resistance was reported in Iran [42] and China [43].

Findings of the present research revealed that the prevalence of resistance to more than one antibiotic agent was high among the *S. aureus* isolates. On the other hand, all isolated bacteria harbored resistance to at least four types of antibiotic agents. Furthermore, the prevalence of resistance to more than eight antibiotic agents was 28.12%. Moreover, the isolates harbored the concurrent presence of two antibiotic resistance genes, particularly *aacA-D* + *blaZ*, *tetK* + *blaZ*, *aacA-D* + *tetK*, *aacA-D* + *gyrA,* and *ermA* + *blaZ* together. A high prevalence of multi-drug resistant *S. aureus* was reported in previous investigations as well [44–46]. However, the literature review showed no report on the prevalence of *gyrA*, *vatC*, *blaZ*, *vatA*, *cat1*, *rpoB*, *dfrA*, *linA*, *vatB*, *msrA*, *aacA-D*, *ermA*, *grlA*, *tetK,* and *tetM* genes among the *S. aureus* bacteria recovered from ready-to-eat food samples. The methylase enzyme modulates the most important mechanism involving resistance to clindamycin, often encoded by the *ermA* gene [47]. The majority of our isolates carried two tetracyclines, two erythromycins, one macrolide, and several streptogramin resistance genes. According to research, the presence of the *tetK* gene on small multicopy plasmids and *tetM* on conjugative transposons makes them spread [48]. Some of the *S. aureus* bacteria harbored the *ermA* gene that is often located on small multicopy plasmids present in many different staphylococcal species. The *ermA* gene is usually carried by transposons, which explains its high prevalence among the *S. aureus* bacteria. The *blaZ* gene encoding beta-lactamase production mainly causes resistance to benzylpenicillin. Our results suggest that *bla*Z may play a major role in the occurrence of resistance to penicillins, but it cannot be used alone as an indicator for penicillin resistance.

## Conclusion

In conclusion, the high prevalence of *S. aureus* in the examined samples, particularly in grilled mushrooms and olivieh salad and a high prevalence of resistance to diverse classes of antibiotic agents and different antibiotic resistance genes were reported in this study. A high prevalence of resistance to penicillin, tetracycline, gentamicin, erythromycin, trimethoprim-sulfamethoxazole, and ciprofloxacin antibiotic agents as well as the presence of *tetK*, *blaZ*, *aacA-D*, *gyrA,* and *ermA* antibiotic resistance genes were reported in the present survey. Furthermore, the high prevalence of multi-drug resistant bacteria and the presence of *aacA-D* + *blaZ*, *tetK* + *blaZ, aacA-D* + *tetK*, *aacA-D* + *gyrA,* and *ermA* + *blaZ* genes together may indicate the leading role of ready-to-eat food samples in the transmission of antibiotic-resistant *S. aureus* to human populations. Accordingly, the use of high-quality raw materials, proper hygienic food-processing conditions, the adequate cooking of food samples, cross-contamination prevention, and antibiotic prescription rendering the outcomes of disk diffusion could diminish the risk of antibiotic-resistant *S. aureus* in the examined samples. Further surveys are recommended to be conducted to illuminate other epidemiological aspects of antibiotic-resistant *S. aureus* in ready-to-eat food samples.

## Methods

### Samples

From April to November 2018, a total of 415 different kinds of ready-to-eat food samples, such as hamburgers (*n* = 75), chicken nuggets (*n* = 70), salad olivieh (*n* = 60), salami (*n* = 60), falafel (*n* = 50), grilled mushrooms (*n* = 50), and Mexican corn (*n* = 50) were randomly collected from the fast-food restaurants of the Tehran province, Iran. Sampling was performed in highly consumed ready-to-eat food samples. According to the low diversity of ready-to-eat food-producing restaurants in Iran, sampling was done in all of them. To this end, simple stratified sampling was performed according to the production volume of each fast food unit. Besides, the samples (100 g) were directly delivered to the Food Hygiene Research Center. In addition, the food samples were transported in cool boxes with ice packs.

### *S. aureus* isolation and identification

As many as 20 g of each collected ready-to-eat food sample was blended with 225 mL of buffered peptone water (Merck, Germany). Next, the solutions were homogenized using a stomacher (Interscience, Saint-Nom, France). Consequently, 5 mL of the produced solution was transferred to 50 mL of Trypticase Soy Broth (TSB, Merck, Germany) supplemented with 10% NaCl and 1% sodium pyruvate, which was then incubated for 18 h at 35 °C. Next, a loopful culture was transferred to the Baird-Parker agar supplemented with an egg yolk tellurite emulsion (Merck, Germany) and incubated at 37 °C for about 24 h. Black shiny colonies surrounded with 2- to 5-mm clear zones were identified based on gram staining, hemolytic activity on the sheep blood agar (Merck, Germany), catalase test, coagulase test (rabbit plasma), oxidase test, OF glucose test, bacitracin sensitivity test (0.04 U), mannitol fermentation on the Mannitol salt agar (Merck, Germany), urease activity, nitrate reduction, phosphatase, deoxyribonuclease (DNase, Merck, Germany) test, Voges-Proskauer (VP) (Merck, Germany) test, and carbohydrate (xylose, sucrose, trehalose and maltose, fructose, lactose, and mannose) fermentation test [13].

### Phenotypic assessment of antibiotic resistance

The phenotypic pattern of antibiotic resistance in *S. aureus* bacteria was investigated using the disk diffusion method on the Mueller–Hinton agar (Merck, Germany). For this purpose, the principles of the Clinical Laboratory Standard Institute (CLSI) were used [49]. Accordingly, various kinds of antibiotic agents, including aminoglycosides (amikacin (30 µg/disk) and gentamicin (10 µg/disk)), fluoroquinolones (levofloxacin (5 µg/disk) and ciprofloxacin (5 µg/disk)), lincosamides (clindamycin (2 µg/disk)), macrolides (erythromycin (15 µg/disk) and azithromycin (15 µg/disk)), penicillins (penicillin (10 µg/disk)), tetracyclines (doxycycline (30 µg/disk) and tetracycline (30 µg/disk)), phenicols (chloramphenicol (30 µg/disk)), folate pathway inhibitors (trimethoprim-sulfamethoxazole (25 µg/disk)) and ansamycins (rifampin (5 µg/disk)) were used (Oxoid, UK). The test was performed using the protocol already explained [49–52]. Besides, *S. aureus* (ATCC 43,300) was used as a quality control organism to determine antimicrobial susceptibility.

### Genotypic assessment of antibiotic resistance

*S. aureus* isolates were sub-cultured on TSB media (Merck, Germany) and incubated for 48 h at 37 °C. Besides, genomic DNA was extracted from bacterial colonies using the DNA extraction kit (Thermo Fisher Scientific, St. Leon-Rot, Germany) according to the manufacturer's instructions. Next, the purity (A260/A280) and concentration of the extracted DNA were checked (NanoDrop, Thermo Scientific, Waltham, MA, USA). In addition, the DNA's quality was assessed on a 2% agarose gel stained with ethidium bromide (0.5 μg/mL) (Thermo Fisher Scientific, St. Leon-Rot, Germany) [53, 54].

Table 5 shows the PCR conditions met for genotypic assessment of antibiotic resistance [48, 55–61]. A programmable DNA thermocycler (Eppendorf Mastercycler 5330, Eppendorf-Nethel-Hinz GmbH, Hamburg, Germany) was used in all PCR reactions. In addition, amplified samples were analyzed by electrophoresis (120 V/208 mA) in a 2.5% agarose gel that was stained with 0.1% ethidium bromide (0.4 µg/ml). Besides, UVI doc gel documentation systems (Grade GB004, Jencons PLC, London, UK) were used to analyze images.

**Table 5:** PCR circumstances used for genotypic assessment of antibiotic resistance [48, 55–61]

Target gene	Primer sequence (5′-3′)	PCR product (bp)	PCR programs	PCR volume (50 µL)
*AacA-D*	F: TAATCCAAGAGCAATAAGGGC R: GCCACACTATCATAACCACTA	227	1 cycle: 94 °C –––––– 5 min	5 µL PCR buffer 10×
*ermA*	F: AAGCGGTAAACCCCTCTGA R: TTCGCAAATCCCTTCTCAAC	190	25 cycle:	1.5 mM Mgcl_2_
			94 °C –––––– 60 s	200 µM dNTP (Fermentas)
			55 °C –––––– 70 s	0.5 µM of each primer F & R
*tetK*	F: GTAGCGACAATAGGTAATAGT R: GTAGTGACAATAAACCTCCTA	360	72 °C –––––– 60 s	1.25 U Taq DNA polymerase (Fermentas)
			1 cycle: 72 °C –––––– 10 min	2.5 µL DNA template
*tetM*	F: AGTGGAGCGATTACAGAA R: CATATGTCCTGGCGTGTCTA	158	1 cycle: 94 °C –––––– 6 min	5 µL PCR buffer 10× 2 mM Mgcl_2_
			34 cycle: 95 °C –––––– 50 s	200 µM dNTP (Fermentas)
			55 °C –––––– 70 s	0.5 µM of each primer F & R
			72 °C –––––– 60 s	1.5 U Taq DNA polymerase (Fermentas)
			1 cycle: 72 °C –––––– 8 min	5 µL DNA template
*msrA*	F: GGCACAATAAGAGTGTTTAAAGG R: AAGTTATATCATGAATAGATTGTCCTGTT	940	1 cycle: 94 °C –––––– 6 min	5 µL PCR buffer 10× 2 mM Mgcl_2_
			34 cycle: 95 °C –––––– 60 s	150 µM dNTP (Fermentas)
			50 °C –––––– 70 s	0.75 µM of each primer F & R
			72 °C –––––– 70 s	1.5 U Taq DNA polymerase (Fermentas)
			1 cycle: 72 °C –––––– 8 min	3 µL DNA template
*linA*	F: GGTGGCTGGGGGGTAGATGTATTAACTGG R: GCTTCTTTTGAAATACATGGTATTTTTCGA	323	1 cycle: 94 °C –––––– 6 min	5 µL PCR buffer 10× 2 mM Mgcl_2_
			30 cycle: 95 °C –––––– 60 s	150 µM dNTP (Fermentas)
			57 °C –––––– 60 s	0.75 µM of each primer F & R
			72 °C –––––– 60 s	1.5 U Taq DNA polymerase (Fermentas)
			1 cycle: 72 °C –––––– 10 min	3 µL DNA template
*blaZ*	F: ACTTCAACACCTGCTGCTTTC R: TGACCACTTTTATCA CAACC	490	1 cycle: 94 °C –––––– 5 min	5 µL PCR buffer 10× 2 mM Mgcl_2_
			30 cycle: 94 °C –––––– 20 s	150 µM dNTP (Fermentas)
			60 °C –––––– 30 s	0.75 µM of each primer F & R
			72 °C –––––– 90 s	1.5 U Taq DNA polymerase (Fermentas)
			1 cycle: 72 °C –––––– 5 min	3 µL DNA template
*cat1*	F: AGTTGCTCAATGTACCTATAACC R: TTGTAATTCATTAAGCATTCTGCC	547	1 cycle: 94 °C –––––– 8 min	5 µL PCR buffer 10× 2 mM Mgcl_2_
			32 cycle: 95 °C –––––– 60 s	150 µM dNTP (Fermentas)
			55 °C –––––– 70 s	0.75 µM of each primer F & R
			72 °C –––––– 2 min	1.5 U Taq DNA polymerase (Fermentas)
			1 cycle: 72 °C –––––– 8 min	3 µL DNA template
*gyrA*	F: AATGAACAAGGTATGACACC R: TACGCGCTTCAGTATAACGC	223	1 cycle: 94 °C –––––– 10 min	5 µL PCR buffer 10X
*grlA*	F: ACTTGAAGATGTTTTAGGTGAT R: TTAGG AAATCTTGATGGCAA	459	25 cycle: 94 °C –––––– 20 s	2 mM Mgcl_2_ 150 µM dNTP (Fermentas)
			52 °C –––––– 20 s	0.75 µM of each primer F & R
			72 °C –––––– 50 s	1.5 U Taq DNA polymerase (Fermentas)
			1 cycle: 72 °C –––––– 5 min	3 µL DNA template
*dfrA*	F: CTCACGATAAACAAAGAGTCA R: CAATCATTGCTTCGTATAACG	201	1 cycle: 94 °C –––––– 2 min	5 µL PCR buffer 10×
			30 cycle:	2 mM Mgcl_2_
			94 °C –––––– 60 s	150 µM dNTP (Fermentas)
			50 °C –––––– 60 s	0.75 µM of each primer F & R
			72 °C –––––– 60 s	1.5 U Taq DNA polymerase (Fermentas)
			1 cycle: 72 °C –––––– 5 min	3 µL DNA template
*rpoB*	F: ACCGTCGTTTACGTTCTGTA R: TCAGTGATAGCATGTGTATC	460	1 cycle: 94 °C –––––– 5 min	5 µL PCR buffer 10×
			32 cycle:	2 mM Mgcl_2_
			94 °C –––––– 60 s	150 µM dNTP (Fermentas)
			56 °C –––––– 45 s	0.75 µM of each primer F & R
			72 °C –––––– 60 s	1.5 U Taq DNA polymerase (Fermentas)
			1 cycle: 72 °C –––––– 10 min	3 µL DNA template

### Statistical analysis

Statistical analysis was performed by SPSS Statistics 21.0 (SPSS Inc., Chicago, IL, USA). Chi-square and Fisher's exact two-tailed tests were performed to assess any significant relationship between the prevalence of *S. aureus* bacteria and their phenotypic and genotypic properties of antibiotic resistance. Besides, *p*-value < 0.05 was considered statistically significant [62].

## Data Availability

All data generated or analyzed throughout this research are included in this published article.

## Abbreviations

*S. aureus*: *Staphylococcus aureus*
PCR: Polymerase chain reaction
SPSS: Statistical package for the social sciences
